# Local Variance-Guided Adaptive Infrared–Thermal Sensor Fusion Framework for Human Target Detection in Smoke-Filled Firefighting Environments

**DOI:** 10.3390/s26175670

**Published:** 2026-09-07

**Authors:** Changyuan Shen, Mingguang Diao, Liyang Wang, Longzhou Li, Rui Wang, Yongkang Chen, Wenji Li

**Affiliations:** 1 School of Artificial Intelligence, China University of Geosciences Beijing, Beijing 100083, China; 2004240038@email.cugb.edu.cn (C.S.); 2104240052@email.cugb.edu.cn (L.W.); 2104240053@email.cugb.edu.cn (L.L.); w1873675302@163.com (R.W.); ykk_chen@163.com (Y.C.); 2 China Aero Geophysical Survey and Remote Sensing Center for Natural Resources, Beijing 100083, China

**Keywords:** infrared–thermal sensor fusion, smoke-filled environments, human target detection, infrared image enhancement, sensor-adaptive fusion, lightweight object detection

## Abstract

**Highlights:**

**What are the main findings?**

- A local variance-guided adaptive infrared–thermal sensor fusion framework is proposed for human target detection in smoke-filled firefighting environments.
- An improved dark channel prior-based infrared desmoking method enhances smoke-degraded infrared representations, while the LVAF strategy enables adaptive integration of infrared structural details and thermal target-related cues.
- The proposed framework achieves an mAP@0.5 of 0.9950 and an mAP@0.5:0.95 of 0.9577 on a dense-smoke infrared–thermal dual-modal dataset.

**What are the implications of the main findings?**

- The proposed framework provides a lightweight perception solution for smoke-filled firefighting scenarios by combining infrared enhancement, multimodal fusion, and YOLO11n-based detection.
- The YOLO11n detection component achieves a detector-stage inference speed of 73.07 FPS with only 2.58 M parameters, showing potential for lightweight firefighting robot perception.

**Abstract:**

Reliable human target detection in smoke-filled environments is essential for firefighting robots and rescue perception systems. However, conventional RGB cameras are severely degraded by dense smoke, while a single infrared or thermal imaging sensor cannot simultaneously provide sufficient structural details and reliable target-related thermal information. To address these challenges, this paper proposes a local variance-guided adaptive infrared–thermal sensor fusion framework for human target detection in smoke-filled environments, aiming to alleviate smoke-induced degradation in multimodal perception through improved infrared representation and adaptive cross-modal information utilization. An improved dark channel prior-based infrared desmoking algorithm is designed, where guided filtering is employed to refine the transmission map, suppress halo artifacts, and enhance infrared image quality. Furthermore, a local variance-guided adaptive fusion strategy is proposed, which utilizes local variance as an information saliency metric to generate pixel-level adaptive modality weights for fusing desmoked infrared and thermal images. In addition, a lightweight YOLO11n detector is adopted to achieve efficient human target recognition while maintaining a favorable balance among detection accuracy, computational cost, and inference efficiency. Experimental results on the self-built dense-smoke dual-modal dataset demonstrate that the proposed framework achieves high detection performance with low model complexity and efficient detector-stage inference. The ablation results demonstrate the contribution of infrared–thermal multimodal fusion to reliable smoke perception and indicate that the proposed local variance-guided adaptive fusion strategy maintains comparable detection accuracy while providing a better detector-stage speed–accuracy balance than fixed-weight fusion. With a low parameter count, the adopted YOLO11n detector shows potential for future deployment on resource-constrained firefighting robotic platforms.

## 1. Introduction

Fire accidents pose significant threats to human life and property safety [1,2], particularly in smoke-filled environments where rapid and reliable perception is essential for effective rescue operations [3]. During firefighting and emergency response, dense smoke severely degrades the performance of conventional visible-light cameras due to light scattering, occlusion, and reduced contrast [4,5]. As a result, firefighting robots equipped with infrared (IR) and thermal imaging sensors have attracted increasing attention because these sensors can provide complementary information under adverse environmental conditions [3,6]. Therefore, achieving accurate and robust human target detection in smoke-filled environments is of great significance for improving the autonomous perception capability of firefighting and rescue systems.

Although infrared and thermal imaging technologies provide advantages over visible-light sensing in smoke scenarios [6,7], reliable human target detection remains challenging. Infrared images can preserve structural and contour information, but their image quality is often degraded by dense smoke interference, resulting in reduced contrast and blurred target boundaries. Thermal images exhibit stronger robustness against smoke occlusion by capturing human radiation characteristics; however, they usually lack sufficient texture and spatial details, which may limit precise localization. Therefore, relying on a single imaging modality cannot simultaneously satisfy the requirements of target visibility, localization accuracy, and environmental robustness. Multimodal infrared–thermal fusion provides a promising solution by combining complementary information from different sensors [7,8,9]. From the perspective of sensor-based perception, the key challenge is not only to combine heterogeneous infrared and thermal sensing information, but also to dynamically adapt modality contributions under asymmetric smoke interference, where different modalities may experience different levels of degradation.

Recent studies have explored image enhancement, multimodal fusion, and deep learning-based detection methods to improve perception performance under challenging conditions [10,11,12]. Image enhancement methods based on physical models, such as dark channel prior and transmission estimation, can improve degraded image visibility by recovering scene information [13,14,15,16,17]. However, most existing enhancement methods are designed for visible-light haze removal and may suffer from inaccurate transmission estimation, halo artifacts, and noise amplification when directly applied to infrared images under dense smoke conditions. Learning-based enhancement methods have improved restoration performance in complex degradation scenarios [18,19,20,21], but they generally require large-scale training data and may lack physical interpretability in smoke-filled environments.

Multimodal fusion methods have also been widely investigated to exploit complementary information from different sensors [12,22]. Traditional fixed-weight fusion methods cannot dynamically adjust modality contributions according to spatially varying image quality, which may result in information redundancy or loss. Adaptive fusion strategies based on gradients, saliency estimation, and attention mechanisms have improved the flexibility of modality selection [23,24]; however, existing fusion strategies rarely consider the spatially varying reliability of infrared and thermal information caused by smoke interference, which limits their effectiveness in human target detection under dense smoke conditions [22,25,26,27]. Therefore, designing a smoke-oriented adaptive fusion strategy that can effectively balance infrared structural information and thermal target saliency remains an important challenge.

Beyond individual enhancement and fusion techniques, practical firefighting applications require perception systems to simultaneously address representation reliability, detection accuracy, inference efficiency, and computational cost [28,29]. Large-scale detection networks usually provide stronger feature representation capability but require substantial computational resources, which limits their deployment on edge devices and mobile rescue platforms in real-time firefighting scenarios. Lightweight detectors reduce computational requirements and improve inference efficiency; however, their robustness may still degrade when the input representations are severely affected by smoke interference [30,31,32]. Therefore, an effective smoke perception framework should establish a coordinated pipeline that connects multimodal representation enhancement with efficient target recognition, enabling robust perception under smoke-induced degradation.

To address the above challenges, this study proposes an infrared–thermal perception framework for human target detection in smoke-filled environments. The proposed framework addresses smoke-induced multimodal perception degradation by jointly improving infrared representation quality, cross-modal information utilization, and lightweight target recognition. Specifically, an improved dark channel prior-based desmoking method is developed to enhance the representation quality of smoke-degraded infrared images by refining the transmission map through guided filtering and introducing a minimum transmission threshold. Furthermore, a local variance-guided adaptive fusion strategy is proposed to achieve pixel-level dynamic weighting between desmoked infrared and thermal images, enabling adaptive exploitation of complementary information from heterogeneous infrared and thermal modalities. Finally, a lightweight YOLO11n-based detector is adopted to achieve efficient human target recognition while maintaining a favorable balance between accuracy and computational cost.

The main contributions of this study are summarized as follows:

1. An infrared representation enhancement method is developed to address smoke-induced degradation in infrared imaging. To address the problems of degraded contrast, inaccurate transmission estimation, and artifact amplification in conventional enhancement methods when applied to smoke-interfered infrared images, an improved dark channel prior-based desmoking algorithm is proposed. Guided filtering is introduced to refine the transmission map while preserving important structural information, and a minimum transmission threshold is incorporated to suppress noise amplification in extremely dense smoke regions. The proposed method improves the quality of infrared representations and provides more informative inputs for subsequent multimodal fusion and human target detection.
2. A local variance-guided infrared–thermal fusion strategy is proposed to address asymmetric information degradation between heterogeneous modalities under smoke interference. To achieve adaptive utilization of heterogeneous sensor information under spatially varying smoke interference, a local variance-based weighting mechanism is designed. Local variance is employed as a modality saliency measurement, while normalization, Gaussian smoothing, and competitive weight allocation are applied to generate pixel-level adaptive fusion weights. The proposed strategy effectively exploits the complementary characteristics of infrared structural information and thermal radiation cues, producing more informative fused representations for human target detection under smoke interference.
3. A lightweight recognition framework is established to achieve efficient human target perception under smoke-degraded multimodal representations. The lightweight YOLO11n detector is initialized with official pretrained weights and fine-tuned on the constructed infrared–thermal dataset for human target detection in practical rescue scenarios. The proposed framework achieves a favorable balance between detection performance, model complexity, and detector-stage inference efficiency, indicating potential for efficient firefighting and rescue perception applications.

## 2. Proposed Infrared–Thermal Sensor Fusion Framework for Human Target Detection

**Figure 1:**
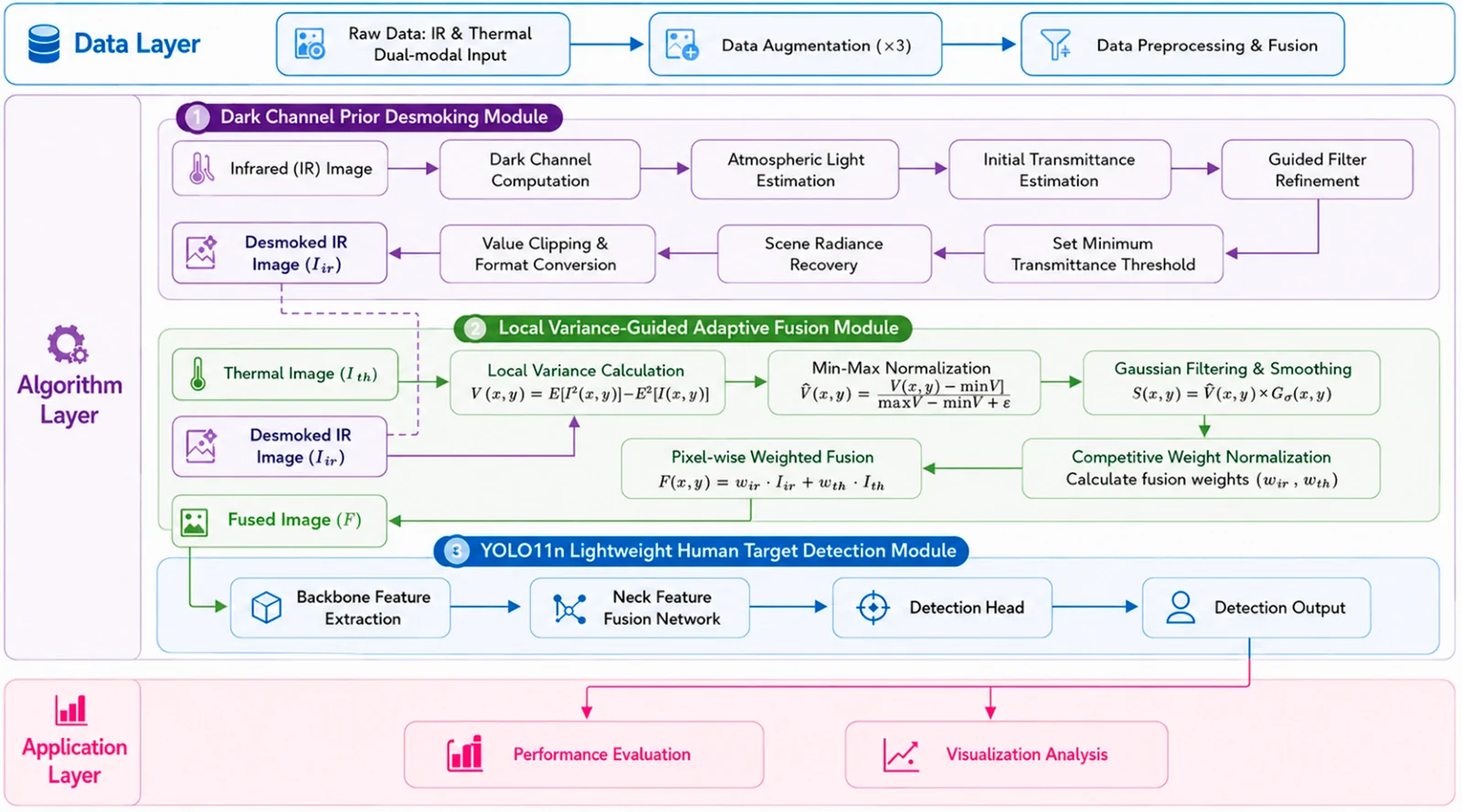
Overall framework of the human target detection method in smoke-filled environments.

The overall framework of the human target detection method in smoke-filled environments is shown in Figure 1. The architecture is broadly divided into a data layer, which illustrates the logical relationships among data, algorithms, and applications. The data layer is responsible for raw data input, enhancement, and preprocessing, ultimately generating fused images. The algorithm layer consists of three core modules: a dark channel prior-based desmoking module, a local variance-guided adaptive fusion module, and a lightweight YOLO11n-based detection module. The application layer comprises evaluation and visualization modules, which are used for performance assessment and visual analysis of the detection results.

### 2.1. Desmoking of Infrared Images in Smoke-Filled Environments

**Figure 2:**
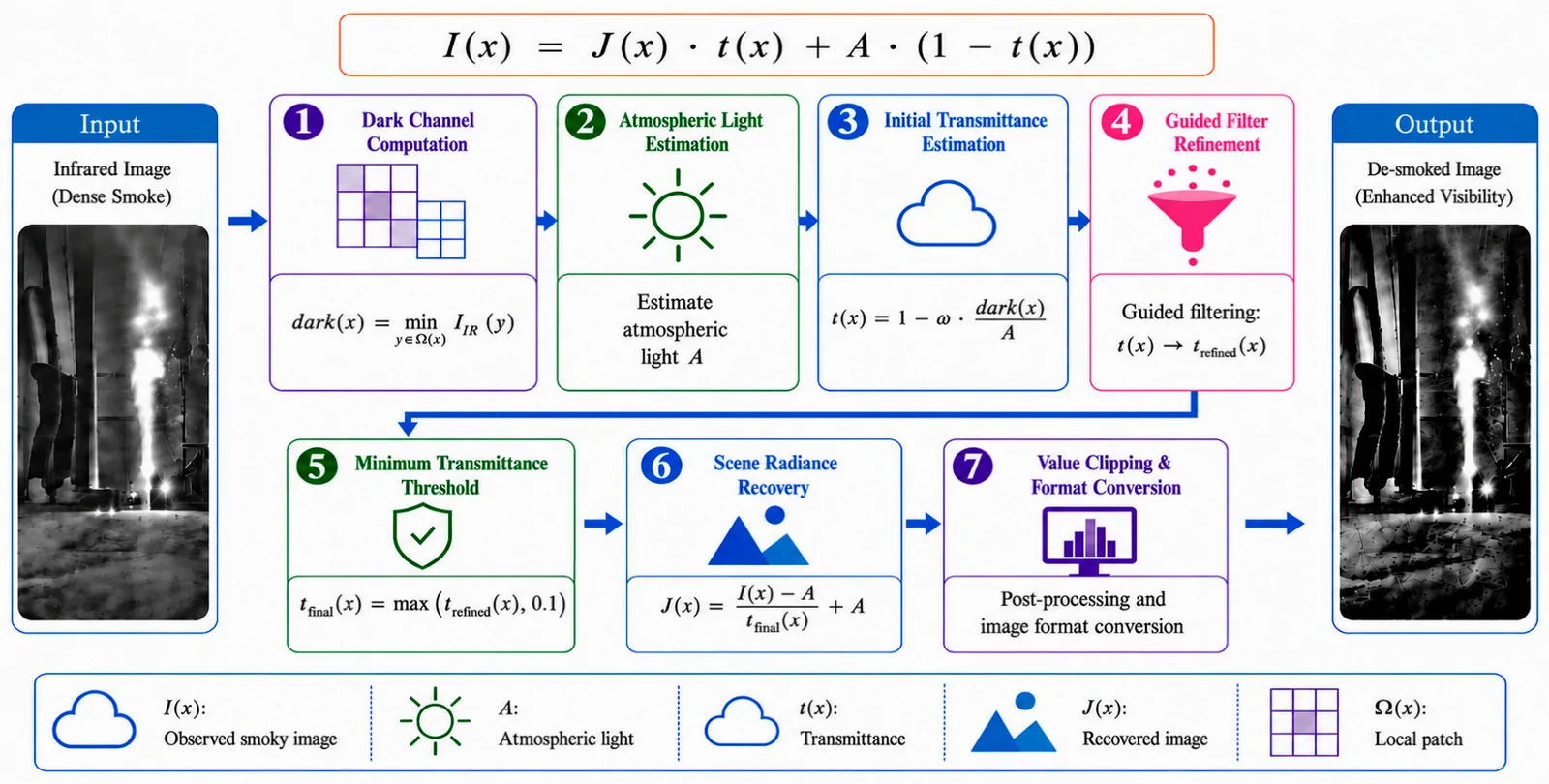
Flowchart of the improved dark channel prior desmoking algorithm.

The dark channel prior has been widely used for single-image haze removal based on atmospheric scattering models [33]. The original Dark Channel Prior (DCP) algorithm uses Soft Matting to refine the transmission map, which has high computational complexity and may produce halo artifacts around object boundaries. To improve its applicability to smoke-filled infrared images, this study introduces two modifications. First, guided filtering [34] is adopted to refine the transmission map and reduce halo artifacts caused by coarse transmission estimation. Second, a minimum transmission threshold is introduced to suppress noise amplification in extremely dense smoke regions, thereby improving the numerical stability of the restored infrared images. While preserving the interpretability of the atmospheric scattering model, the improved DCP module is better suited for smoke-oriented infrared image enhancement before multimodal fusion. The flowchart of the improved dark channel prior desmoking algorithm used in this paper is shown in Figure 2.

The theoretical foundations of the improved DCP-based desmoking algorithm include the atmospheric scattering model, dark channel prior, transmission estimation, transmission refinement, and scene recovery.

In smoke-filled environments, the atmospheric scattering model provides a physical basis for describing image degradation caused by smoke scattering and attenuation. By estimating the transmission map and scene radiance, the model can be used to enhance smoke-degraded infrared images and recover clearer structural information for subsequent human target detection. In this model, $I\left(x\right)$ represents the observed smoke-degraded infrared image, $J\left(x\right)$ denotes the restored infrared image, $t\left(x\right)$ represents the transmission map describing the attenuation of infrared radiation caused by smoke, and A represents the estimated atmospheric light component under smoke conditions. In this study, A is estimated adaptively. Specifically, the top 0.1% pixels with the highest dark channel responses are selected, and the corresponding pixel values in the input image are averaged to obtain the atmospheric light estimation.

(1) I(x)=J(x)·t(x)+A·(1−t(x))

In dense smoke-filled environments, directly applying the original DCP algorithm may lead to unstable transmission estimation because infrared images often exhibit different intensity distributions from natural RGB images. Therefore, in this study, DCP is not used as an independent desmoking solution. Instead, it is adopted as a physical-model-based transmission estimation module and further improved through guided filtering and minimum transmission thresholding.

The dark channel prior was originally developed for RGB natural images, where the minimum operation is performed across multiple color channels. In this study, the original multi-channel minimum operation is adapted to grayscale infrared images by performing local minimum estimation only on the infrared intensity domain. However, infrared images used in smoke-filled environments are typically single-channel intensity images without explicit color information. This modification preserves the underlying assumption that smoke scattering causes intensity attenuation and transmission degradation, while avoiding the dependence on RGB color channels. Here, $\Omega \left(x\right)$ denotes a local patch centered at pixel x:

(2) $$dark\left(x\right)=\underset{y\in \Omega \left(x\right)}{\min}I_{IR}(y)$$

Transmission estimation is divided into three stages: initial transmission estimation, transmission refinement, and final transmission thresholding.

The initial transmission map is estimated based on the adapted dark channel prior. Here, ω is the desmoking factor, which controls the degree of smoke attenuation compensation and avoids excessive restoration:

(3) $$t\left(x\right)=1-\omega \cdot \frac{dark(x)}{A}$$

Transmission refinement is performed using guided filtering to smooth the initial transmission map while preserving important structural edges. The initial transmission map t(x) may contain block artifacts and rough boundaries due to the patch-based estimation of the dark channel prior. If it is directly used for scene recovery, local discontinuities may lead to halo artifacts around object contours and background edges. Therefore, guided filtering is introduced to refine the initial transmission map.

In this study, the original smoke-degraded infrared image is converted to grayscale and used as the guidance image, while the initial transmission map is used as the filtering input. The grayscale infrared image is selected as the guidance image instead of the thermal image because the transmission map is estimated from the infrared modality. Using the same modality as guidance helps preserve structural consistency between the transmission map and the infrared image to be restored. In contrast, thermal images mainly reflect temperature radiation rather than infrared texture distribution. If a thermal image is used as the guidance image, cross-modal structural mismatch may be introduced into the transmission refinement process, which could distort infrared texture details and human contours. Therefore, the grayscale infrared image is adopted as the guidance image for transmission refinement. After guided filtering, the refined transmission map $t_{\mathrm{refined}}(x)$ is obtained.

Finally, to prevent noise amplification in extremely dense smoke regions where the estimated transmission approaches zero, a minimum transmission threshold is introduced. In this study, the minimum transmission value is set to 0.1. This threshold avoids excessive amplification of high-frequency noise during scene recovery and yields the final transmission map $t_{\mathrm{final}}(x)$:

(4) $$t_{final}\left(x\right)=max(t_{refined}\left(x\right),0.1)$$

In the scene recovery stage, the final desmoked infrared image is recovered by substituting the refined transmission map $t_{\mathrm{final}}(x)$ into the inverse atmospheric scattering model. Since the atmospheric light component and transmission map are estimated in the infrared intensity domain, the recovered image is consistent with the adapted DCP formulation for smoke-degraded infrared imaging.

(5) $$J\left(x\right)=\frac{I\left(x\right)-A}{t_{final}(x)}+A$$

### 2.2. Multimodal Adaptive Fusion in Smoke-Filled Environments

Traditional multimodal fusion methods, such as fixed-weight averaging and simple gradient-based weighting, often suffer from rigid weight allocation and unstable local responses in smoke-filled environments. Fixed-weight fusion cannot adaptively adjust the contribution of each modality according to spatially varying image characteristics, while gradient-based weighting may be sensitive to sensor noise and smoke-induced local fluctuations. These limitations may lead to blurred object boundaries, amplified background noise, or local artifacts in fused images, thereby affecting subsequent human target detection. To address these issues, this study proposes a local variance-guided adaptive fusion strategy based on local information saliency estimation.

The core idea of the proposed fusion strategy is to assign pixel-level modality weights according to the local information characteristics of desmoked infrared and thermal images. Specifically, local variance is employed as a local information descriptor to characterize spatial variations in texture, contour structures, and thermal responses within each modality. It should be noted that local variance is not regarded as an explicit sensor reliability estimator, but rather as a spatial information descriptor for estimating modality contributions. After local information estimation, Min–Max normalization is applied to eliminate scale inconsistency between the two modalities. Gaussian filtering is then used to smooth the saliency maps and suppress isolated noisy responses. Finally, a competitive weight normalization strategy is adopted to generate complementary modality-specific fusion weights for infrared and thermal images. Through this process, the proposed method adaptively preserves infrared structural details and thermal target-related information in smoke-filled scenes.

**Figure 3:**
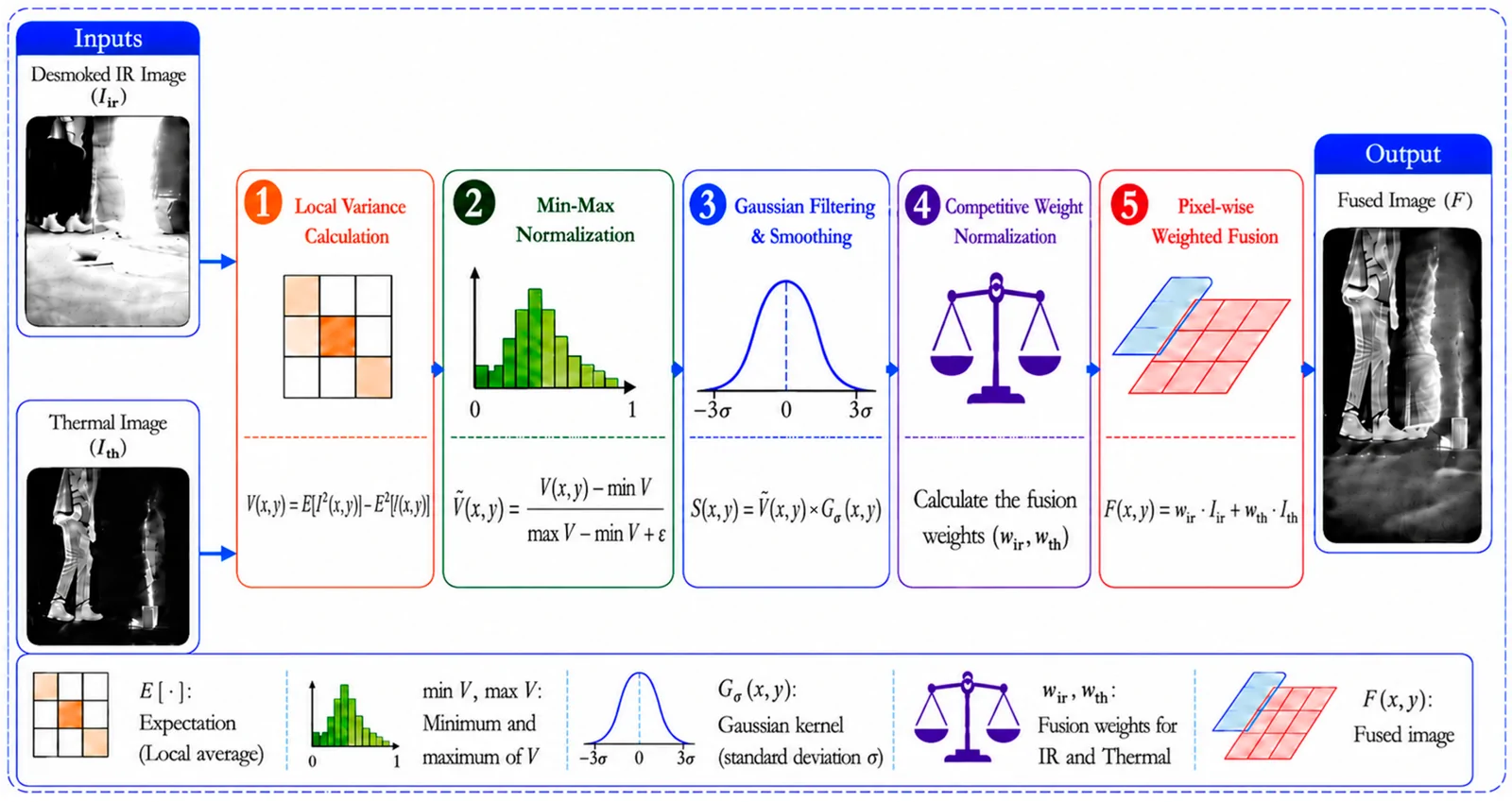
Flowchart of the local variance-guided adaptive fusion algorithm.

The flowchart of the local variance-guided adaptive fusion algorithm used in this paper is shown in Figure 3.

Local variance definition. Local variance is used to measure the local information richness of each modality. In smoke-occluded regions, infrared images may suffer from contrast degradation due to smoke scattering, while thermal images can still preserve human thermal radiation cues. In clearer structural regions, infrared images may retain more contour and texture details. Therefore, local variance provides a simple and interpretable saliency measure for adaptive modality weighting:

(6) $$V\left(x,y\right)=E\left[I^{2}\left(x,y\right)\right]-E^{2}[I(x,y)]$$

Here, E[⋅] denotes the mean operation within a local k×k=7×7 window. The 7×7 window is selected as a practical compromise between local saliency sensitivity and noise suppression. A smaller window may be more sensitive to isolated sensor noise and smoke-induced local fluctuations, whereas a larger window may oversmooth local target boundaries and weaken small-scale human contour information. Therefore, the 7×7 setting is adopted to maintain local structural sensitivity while reducing unstable pixel-level variations.

Min–Max normalization. Since the infrared and thermal modalities may have different intensity ranges and variance distributions, Min–Max normalization is applied to map the local variance responses of each modality into the range [0,1]:

(7) $$\hat{V}\left(x,y\right)=\left[V\left(x,y\right)-minV\right]/\left[maxV-minV+\varepsilon \right]$$

where $\varepsilon =10^{-8}$ is a small constant used to avoid division by zero and numerical instability when the variance range is close to zero. This normalization step allows the two modalities to be compared under a unified scale and prevents one modality from dominating the fusion process solely due to differences in numerical magnitude.

Gaussian filtering. After normalization, Gaussian filtering is applied to smooth the saliency maps and generate spatially continuous fusion weight maps:

(8) $$S\left(x,y\right)=\hat{V}\left(x,y\right)\times G_{\sigma }(x,y)$$

where $G_{\sigma }$ denotes a Gaussian smoothing operator, and $S\left(x,y\right)$ represents the smoothed local information response obtained from the normalized local variance map. In this study, σ is set to 5, and the kernel size is automatically determined by the implementation. Gaussian smoothing suppresses isolated noisy responses caused by sensor noise or local smoke disturbances and reduces abrupt pixel-level weight variations, thereby producing smoother fusion results and reducing local artifacts.

Competitive weight normalization. To obtain complementary modality weights, the smoothed local information responses of the infrared and thermal modalities are normalized through a competitive weighting mechanism:

(9) $$w_{ir}=S_{ir}/(S_{ir}+S_{th}+\varepsilon )$$

(10) $$w_{th}=S_{th}/(S_{ir}+S_{th}+\varepsilon )$$

where $w_{ir}$ and $w_{th}$ denote the fusion weights assigned to the infrared and thermal modalities, respectively. The constant $\varepsilon =10^{-8}$ is added to the denominator to avoid numerical instability when the saliency responses of both modalities are close to zero. After normalization, the dual-modality fusion weights approximately satisfy $w_{ir}$ + $w_{th}$ ≈ 1, enabling pixel-level complementary fusion between infrared structural details and thermal radiation cues.

Final image fusion. The final fused image is obtained by weighted summation of the desmoked infrared image and the thermal image:

(11) $$F\left(x,y\right)=w_{ir}\cdot I_{ir}+w_{th}\cdot I_{th}$$

where $I_{ir}$ and $I_{th}$ represent the desmoked infrared image and the thermal image, respectively. Through pixel-wise adaptive weighting, the fused image can retain both the structural information of infrared imaging and thermal target-related information. This provides a more informative input representation for subsequent human target detection, which is further evaluated through the comparative and ablation experiments in Section 3.5.

### 2.3. Lightweight Human Target Detection Framework in Smoke-Filled Environments

After infrared enhancement and adaptive multimodal fusion, an efficient detection model is required to accurately localize human targets while maintaining real-time inference capability. Therefore, this study adopts YOLO11n as the final detector to perform human target detection on the fused infrared–thermal images.

YOLO11n is selected as the detection backbone based on the following considerations. First, compared with larger-scale detector variants, YOLO11n provides sufficient feature representation capability for the single-class human target detection task while significantly reducing the number of parameters and computational complexity. Second, its lightweight architecture enables efficient inference under limited computational resources, which is beneficial for real-time monitoring in smoke-filled firefighting scenarios. Third, the compact model size provides potential for future deployment on resource-constrained embedded platforms.

It should be noted that YOLO11n is directly adopted without structural modification in this study. The main contribution of the proposed framework lies in the smoke-oriented infrared enhancement and local variance-guided infrared–thermal fusion strategy before detection, which aims to construct more discriminative representations under dense smoke interference. YOLO11n serves as an efficient detector to evaluate whether the enhanced fused representation can provide sufficient discriminative information for human target localization under dense smoke conditions.

During training, the YOLO11n model is initialized with official pretrained weights and fine-tuned on the constructed infrared–thermal human detection dataset. The model is trained using unified experimental settings, including the same input resolution, optimizer configuration, batch size, and training strategy described in Section 3.2. The detection results obtained from YOLO11n are further analyzed through comparative experiments and ablation studies to verify the effectiveness of the proposed preprocessing and fusion modules.

## 3. Results Analysis of the Human Target Detection Method in Smoke-Filled Environments

This section is organized into dataset description, experimental settings, evaluation metrics, training convergence analysis, comparative experiments, and qualitative visualization analysis, in order to verify the effectiveness and practical applicability of the proposed framework.

### 3.1. Dataset Description

The dataset used in this study is a dense-smoke dual-modal human target detection dataset collected from an industrial firefighting simulation scenario and provided by an industrial partner. The dataset is designed for human target detection under smoke-occluded firefighting and rescue environments. It consists of two synchronized imaging modalities, including near-infrared (IR) images and thermal images, which provide complementary information regarding target contours, thermal radiation characteristics, and background interference under complex smoke conditions.

The dataset was collected under simulated smoke-filled environments, where human targets appeared with different postures and spatial positions. The IR and thermal images were temporally and spatially aligned to ensure reliable correspondence during the multimodal image fusion process. All human targets were manually annotated in the YOLO format, where class 0 represents the human target category.

The original images have a resolution of 1920 × 1080 pixels. Due to the data confidentiality agreement with the industrial partner, detailed sensor model information and acquisition parameters are not publicly available. Nevertheless, the processed infrared–thermal image pairs and corresponding annotation files generated in this study have been released through the DOI link provided in the Data Availability Statement to support reproducibility and facilitate further research on smoke-filled human target detection.

The dataset was divided into training and validation subsets after image alignment and annotation. For each modality, the dataset contains 2245 training images and 942 validation images. To improve model generalization, data augmentation was applied only to the training set after dataset partitioning. The augmentation strategies include horizontal flipping, vertical flipping, and brightness enhancement (+30%). These operations are designed to simulate variations in viewpoint and illumination conditions while preserving the original smoke interference characteristics. In addition, an extended validation protocol was constructed separately for robustness evaluation, as described in Section 3.4. The extended validation samples were not used for model training.

### 3.2. Experimental Settings

The proposed method was implemented based on the Ultralytics YOLO framework. The YOLO11n detector was initialized with official pretrained weights (yolo11n.pt) and fine-tuned on the constructed infrared–thermal fusion dataset. Transfer learning was adopted instead of training from scratch to improve convergence and alleviate overfitting caused by the relatively limited number of training samples. During fine-tuning, all model parameters were updated, and no backbone layers were frozen.

All experiments were conducted on a computer equipped with an NVIDIA GeForce RTX 5060 Laptop GPU with 8 GB memory and an AMD Ryzen 9 8945HX processor under the Windows operating system (Version 22H2, Build 19045). The input images were resized to 640 × 640 pixels using the default Letterbox strategy. The model was trained for 100 epochs with a batch size of 16 using the AdamW optimizer (implemented in PyTorch 2.4.0 and Ultralytics 8.3.0). The initial learning rate was set to $1\times 10^{-3}$, with a momentum of 0.937 and a weight decay of $5\times 10^{-4}$. A cosine annealing learning rate strategy was adopted, where the learning rate gradually decayed according to the final learning rate factor of 0.01. Mixed-precision (AMP) training was enabled to improve computational efficiency. An early stopping strategy with a patience of 50 epochs was employed based on validation performance to prevent overfitting.

During training, online data augmentation was applied to improve model robustness. Considering the imaging characteristics of infrared and thermal modalities in smoke-filled environments, several augmentation operations that may introduce unrealistic variations were disabled, including rotation, shear transformation, perspective transformation, and MixUp. Offline augmentations, including horizontal flipping, vertical flipping, and brightness adjustment, were retained to improve robustness against viewpoint and illumination variations while maintaining the physical consistency of smoke-degraded multimodal images.

### 3.3. Evaluation Metrics

The evaluation metrics adopted in this study include Intersection over Union (IoU), Recall, Miss Rate (MR), Precision, F1-score, Average Precision (AP), and Mean Average Precision (mAP). The corresponding formulas are given as follows:

(12) $$IoU=\frac{\left|B_{p}\cap B_{g}\right|}{\left|B_{p}\cup B_{g}\right|}$$

(13) $$Precision=\frac{TP}{TP+FP}$$

(14) $$Recall=\frac{TP}{TP+FN}$$

(15) $$MR=1-Recall=\frac{FN}{TP+FN}$$

(16) $$F1=\frac{2\times Precision\times Recall}{Precision+Recall}$$

(17) $$AP=\sum (R_{n}-R_{n-1})P_{n}$$

(18) $$mAP=\frac{1}{C}\sum_{i=1}^{C}{AP}_{i}$$

where TP, FP, and FN represent the numbers of true positives, false positives, and false negatives, respectively. Bp and Bg denote the predicted and ground-truth bounding boxes. C represents the number of object categories.

Intersection over Union (IoU): IoU is the fundamental criterion for determining whether a predicted bounding box matches the corresponding ground-truth object. It is defined as the ratio between the intersection area and the union area of the predicted bounding box $B_{p}$ and the ground-truth bounding box $B_{g}$. A prediction is regarded as a true positive when the IoU exceeds the specified threshold. In this study, IoU = 0.5 is adopted for mAP@0.5, while IoU thresholds ranging from 0.50 to 0.95 with a step size of 0.05 are adopted for mAP@0.5:0.95.

Precision: Precision measures the proportion of correctly detected human targets among all predicted bounding boxes. A higher Precision indicates fewer false positive detections and better reliability of the detection system.

Recall: Recall measures the proportion of ground-truth human targets that are successfully detected by the proposed method. In firefighting rescue scenarios, missing trapped persons may result in severe consequences; therefore, Recall is considered one of the most important evaluation metrics.

Miss Rate (MR): MR is defined as the complement of Recall. It reflects the proportion of human targets that fail to be detected by the system. A lower MR indicates fewer missed detections and improved safety performance in rescue applications.

F1-score: The F1-score is the harmonic mean of Precision and Recall. It provides a balanced evaluation by simultaneously considering false positives and false negatives.

Average Precision (AP): AP summarizes the Precision–Recall curve by integrating precision over different recall levels. It evaluates the detection performance by considering the trade-off between precision and recall under different confidence thresholds.

Mean Average Precision (mAP): mAP is calculated by averaging AP values across all object categories. Since this study focuses on a single human target category, mAP is numerically equivalent to the AP of this category.

Following the COCO evaluation protocol, two variants of mAP are adopted:

1. mAP@0.5 evaluates detection performance using an IoU threshold of 0.5.
2. mAP@0.5:0.95 calculates the average AP over IoU thresholds from 0.50 to 0.95 with an interval of 0.05, providing a stricter evaluation of localization accuracy.

### 3.4. Training Convergence Analysis

This section analyzes the convergence behavior of the proposed method during training and evaluates the stability of the optimization process under smoke-filled human target detection scenarios. The original validation set contains 942 synchronized IR/Thermal image pairs. To further evaluate the robustness of the proposed method against practical imaging variations, an extended validation protocol was constructed by applying horizontal flipping, vertical flipping, and brightness enhancement (+30%) to the validation samples. These operations simulate viewpoint variations caused by different observation angles and illumination intensity fluctuations under smoke interference conditions. After extension, the validation set contains 3768 fused images with 3772 annotated human target instances. All quantitative performance results reported in this study are calculated based on this extended validation set.

**Figure 4:**
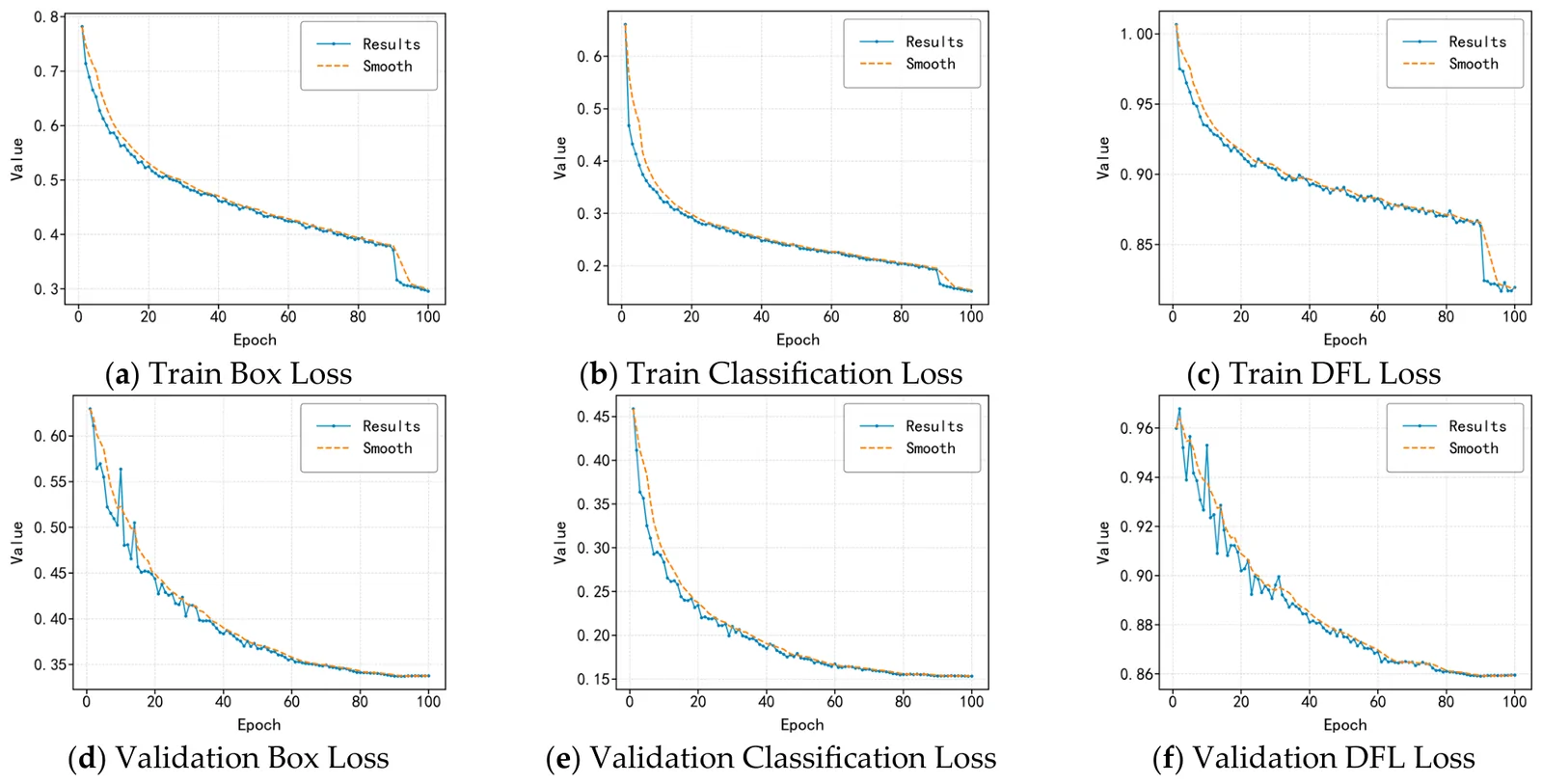
Loss convergence curves during model training.

Figure 4 illustrates the convergence curves of training and validation losses over 100 epochs, where the blue curves represent the original loss values and the orange curves indicate the smoothed trends. The bounding box regression loss, classification loss, and distribution focal loss on both training and validation sets exhibit consistent decreasing trends throughout the training process. During the early training stage, all loss functions decrease rapidly as the model learns discriminative features from the infrared–thermal fusion images. In the later stage, the loss values gradually stabilize, indicating effective optimization. Moreover, no obvious divergence is observed between the training and validation losses, suggesting that the proposed model avoids significant overfitting during fine-tuning.

**Figure 5:**
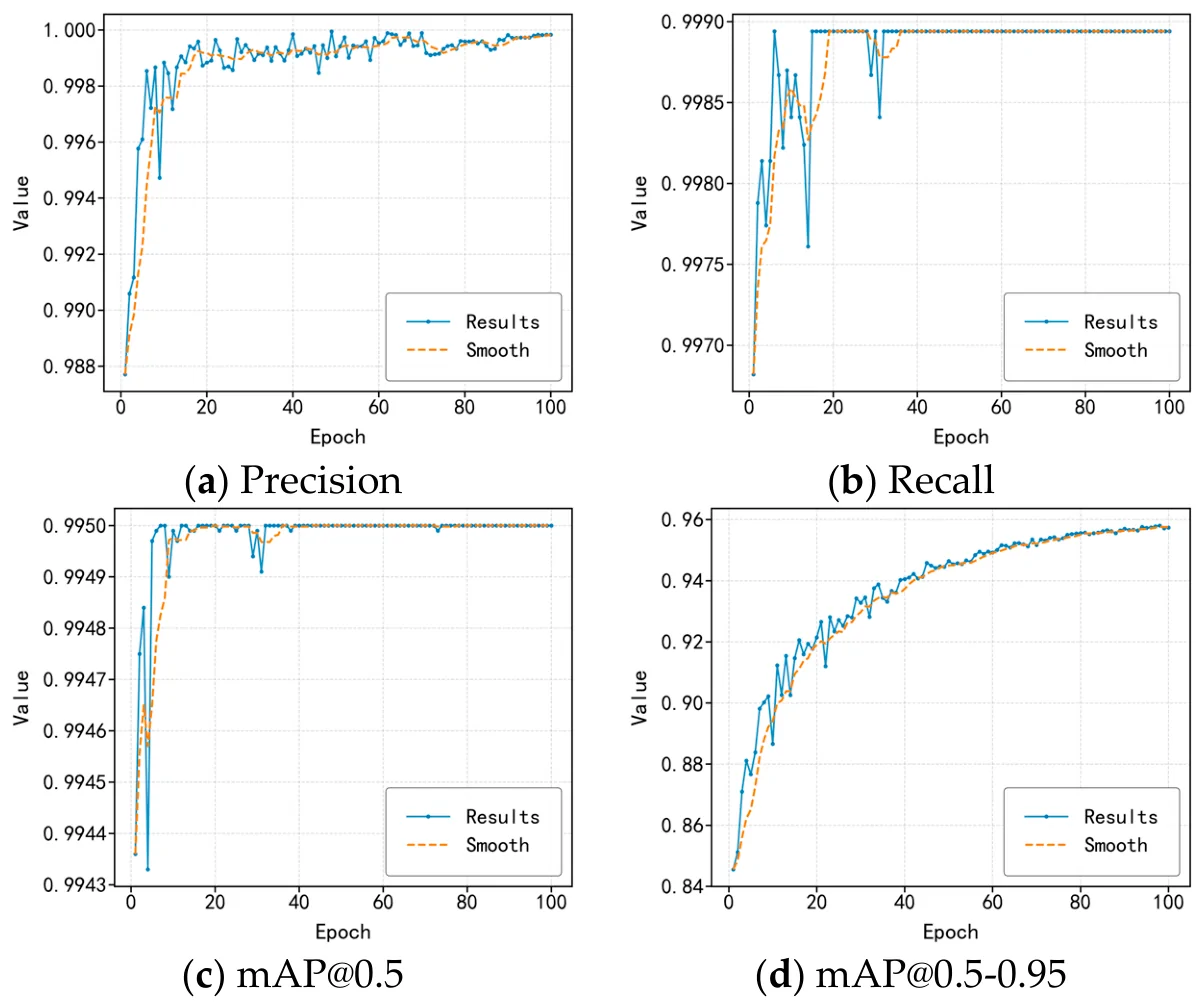
Convergence curves of accuracy metrics during model training.

Figure 5 presents the convergence curves of validation performance metrics automatically recorded by the YOLO framework during training, including Precision, Recall, mAP@0.5, and mAP@0.5:0.95. Precision and Recall increase rapidly during the early training stage and gradually stabilize, indicating that the model effectively learns discriminative features from the infrared–thermal fusion images. Meanwhile, both mAP@0.5 and mAP@0.5:0.95 continuously improve throughout training and converge in the later stage, demonstrating stable optimization behavior and reliable localization capability under smoke-filled conditions.

### 3.5. Comparative Experiments

This section conducts comparative experiments from two perspectives: representative YOLO-based detector comparison and ablation analysis of preprocessing and fusion strategies. All experiments were conducted under the same hardware platform and identical training hyperparameters to ensure fairness and reproducibility.

#### 3.5.1. Detector Selection Under the Proposed Infrared–Thermal Representation

Table 1 evaluates the suitability of different YOLO-based detectors when applied to the proposed infrared–thermal fusion representation. The compared models include different YOLO variants (YOLOv8n, YOLOv8s, YOLO11s, and YOLO11m), representing lightweight and medium-scale detectors with different model capacities. To ensure a fair comparison, all detectors were initialized with their official pretrained weights and fine-tuned on the same infrared–thermal fusion dataset using identical training hyperparameters. All models received the same fused infrared–thermal images generated by the proposed preprocessing and fusion pipeline as input, and only the detector architecture was changed. The inference speed was measured on the same hardware platform with a batch size of 1 and only considered the detector-stage inference time of each detection model. The preprocessing time of infrared desmoking and multimodal fusion was excluded because all detectors shared the same fused input images.

As shown in Table 1, all compared detectors achieve a similar mAP@0.5 of 0.9950, indicating that the proposed infrared–thermal fusion representation provides sufficiently discriminative features for human target detection under the constructed smoke-filled dataset. The limited variation in mAP@0.5 among different YOLO variants suggests that the proposed fusion representation plays an important role in providing effective feature representations for subsequent detection. Compared with YOLO11m, which contains 20.03 M parameters, the selected YOLO11n detector requires only 2.58 M parameters while maintaining the same mAP@0.5. This demonstrates that a lightweight detector can effectively utilize the proposed infrared–thermal fusion representation for human target detection under smoke-filled conditions.

In terms of strict localization accuracy, the selected YOLO11n-based detector achieves an mAP@0.5:0.95 of 0.9577, showing only a marginal difference compared with YOLOv8s and YOLO11m despite its much smaller model scale. Considering its significantly reduced parameter size, this result indicates that YOLO11n provides a favorable balance between localization accuracy and model complexity when used as the detector component of the proposed framework.

Regarding detector-stage inference efficiency, YOLOv8n achieves the highest FPS among the compared models. The selected YOLO11n detector has fewer parameters than YOLOv8n, but its inference speed is slightly lower. This is reasonable because inference speed is not determined only by the number of parameters, but is also affected by network structure, operator types, feature aggregation paths, and GPU execution efficiency. Overall, the selected YOLO11n detector provides a competitive speed–accuracy–complexity trade-off as the detection component of the proposed infrared–thermal perception framework.

Although the comparison mainly focuses on lightweight YOLO-family detectors, the objective of this experiment is not to establish the superiority of YOLO11n over other object detection architectures, but to verify whether a lightweight detector can effectively exploit the proposed infrared–thermal fusion representation under smoke-filled conditions.

#### 3.5.2. Ablation Study of Preprocessing and Multimodal Fusion Strategies

To further analyze the contribution of infrared enhancement and infrared–thermal multimodal fusion, four ablation configurations were designed, as shown in Table 2. In all configurations, the YOLO11n detector architecture was kept unchanged. For each configuration, an independent YOLO11n detector was fine-tuned using the corresponding input setting, while the training hyperparameters, hardware platform, and evaluation protocol were kept identical. The FPS values in Table 2 represent the average inference throughput of the YOLO11n detector with a batch size of 1. The measurement includes image loading, resizing, normalization, forward propagation, and post-processing operations within the Ultralytics inference pipeline. The offline desmoking and fusion generation processes were excluded because all configurations used pre-generated input images during detector inference evaluation.

The four configurations are defined as follows:

- Full Method: Desmoked IR + Local Variance-Guided Adaptive Fusion (LVAF) + YOLO11n, which represents the complete proposed framework.
- No Desmoke: Raw IR without desmoking + Local Variance-Guided Adaptive Fusion (LVAF) + YOLO11n, which is used to evaluate the influence of the desmoking module.
- No Fusion: Desmoked IR only + YOLO11n, where the thermal modality is removed to evaluate the contribution of infrared–thermal multimodal fusion.
- Simple Fusion: Desmoked IR + fixed-weight fusion with α = 0.5 + YOLO11n, where the proposed LVAF strategy is replaced by a fixed-weight averaging strategy.

Compared with the No Fusion configuration, the Full Method improves mAP@0.5:0.95 from 0.9205 to 0.9577 and increases Recall from 0.9958 to 0.9989. This demonstrates the effectiveness of infrared–thermal multimodal fusion for improving human target detection performance under smoke-filled conditions. Thermal images provide complementary information related to human thermal radiation, which helps preserve target-related cues and reduce missed detections when infrared structural information is degraded by dense smoke. The improvement in Recall is particularly meaningful for smoke-filled rescue scenarios, where missed detections of human targets may lead to severe consequences.

Compared with the Simple Fusion configuration, the Full Method achieves the same mAP@0.5 and Recall, while showing only marginal differences in mAP@0.5:0.95 and Precision. Under the same YOLO11n detection framework, the Full Method achieves a higher measured detector-stage inference throughput (72.79 FPS versus 51.16 FPS). This result indicates that the proposed LVAF strategy provides comparable detection performance while achieving a better overall speed–accuracy balance within the same YOLO11n detection framework.

Compared with the No Desmoking configuration, the Full Method maintains the same Recall and comparable detection performance, while achieving a higher measured detector-stage inference throughput. It should be noted that the desmoking module does not independently improve strict localization accuracy under the current dataset. Instead, its contribution mainly lies in improving infrared representation quality and providing more informative inputs for subsequent multimodal fusion. These results suggest that the overall effectiveness of the proposed framework is achieved through the complementary roles of infrared representation enhancement and infrared–thermal multimodal fusion.

Overall, the ablation results show that the Full Method does not achieve the highest value in every individual evaluation metric, but it provides a favorable speed–accuracy trade-off among detection performance, recall, and detector-stage inference efficiency. This balance is particularly important for smoke-filled rescue scenarios, where both low miss rate and real-time response are required.

### 3.6. Qualitative Visualization Analysis

This section presents qualitative visualization results to intuitively demonstrate the performance of the proposed method for human target detection in smoke-filled environments. The analysis focuses on three aspects: detection results under different validation transformations, fusion weight visualization, and Precision–Recall (PR) curve analysis.

#### 3.6.1. Detection Results Under Different Extended Validation Transformations

**Figure 6:**
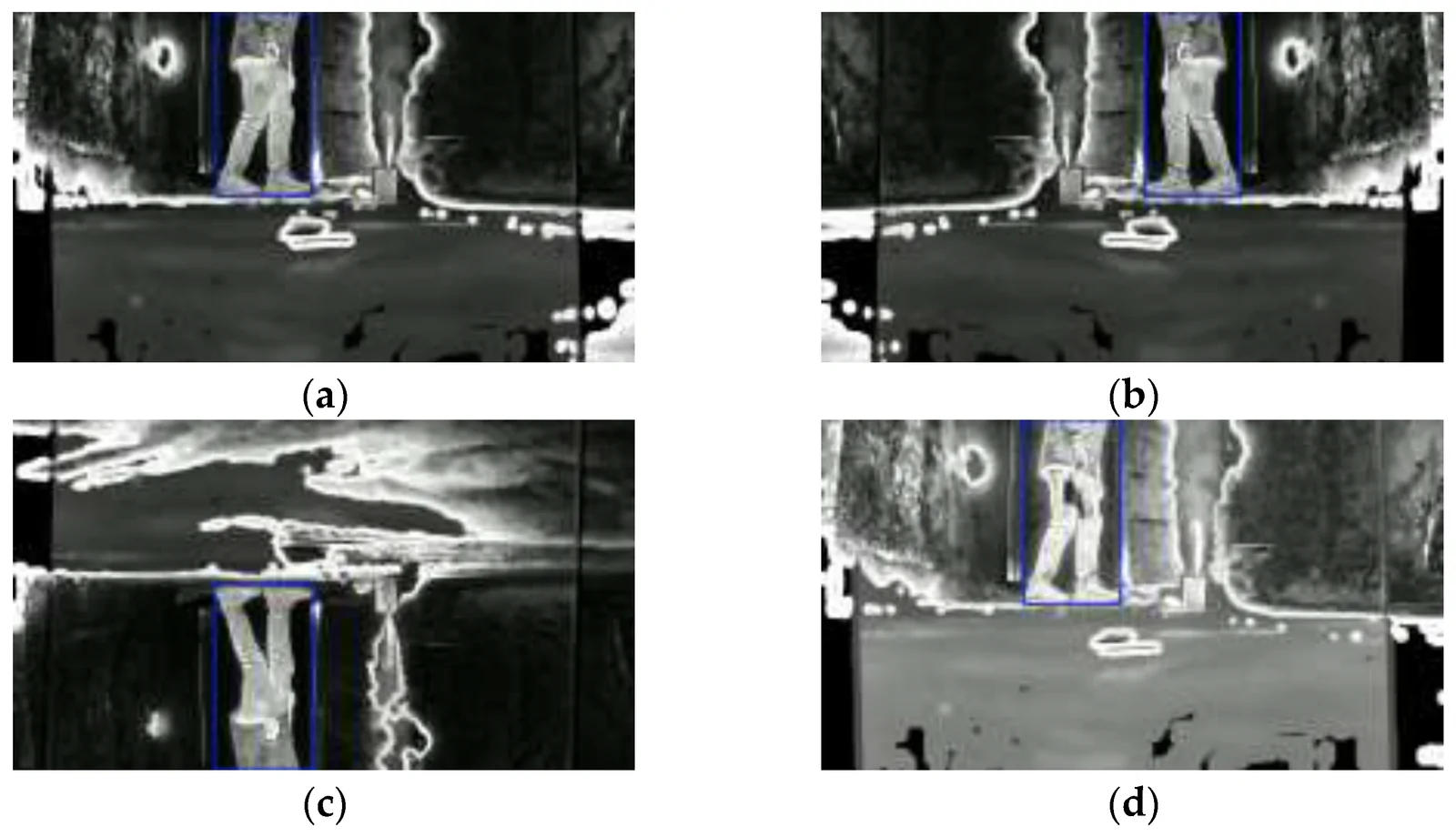
Detection results of the same validation sample under different extended validation transformations. (**a**) Original. (**b**) Horizontal flipping. (**c**) Vertical flipping. (**d**) Brightness enhancement.

Figure 6 presents representative detection results of the same validation sample under different extended validation transformations. In each subfigure, the blue bounding boxes indicate the predicted human target locations.

As shown in Figure 6a–d, the proposed method can stably localize the human target under different image transformations. Even when the input image is flipped or its brightness is enhanced, the predicted bounding boxes remain highly consistent with the target position. These results indicate that the proposed framework maintains robust detection performance under viewpoint variations and illumination intensity disturbances, which is consistent with the design objective of the extended validation protocol.

#### 3.6.2. Modality Fusion Weight Visualization

**Figure 7:**
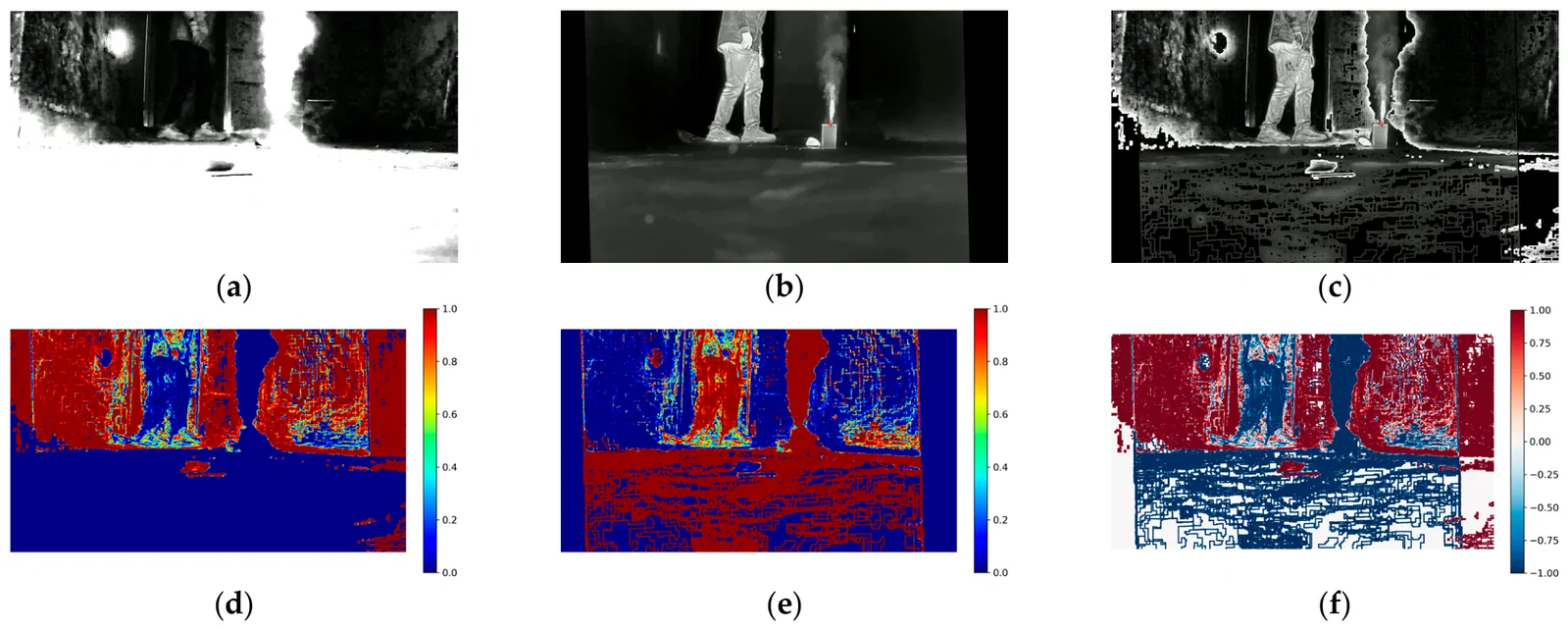
Visualization of modality fusion weights. (**a**) IR Image (Desmoked). (**b**) Thermal Image. (**c**) Fused Image. (**d**) IR Fusion Weight. (**e**) Thermal Fusion Weight. (**f**) Fusion Weight Difference (IR—Thermal).

To further interpret the detection result shown in Figure 6a, Figure 7 visualizes the corresponding modality fusion weights calculated by the Local Variance-Guided Adaptive Fusion (LVAF) module for the same scene sample. Specifically, Figure 7a shows the desmoked infrared image, Figure 7b shows the thermal image, Figure 7c shows the fused image, Figure 7d shows the infrared fusion weight map, Figure 7e shows the thermal fusion weight map, and Figure 7f shows the fusion weight difference map (IR−Thermal). In the fusion weight difference map, red regions indicate higher infrared weights, blue regions indicate higher thermal weights, and intermediate colors indicate similar contributions from the two modalities.

From the qualitative visualization, it can be observed that the LVAF module exhibits adaptive modality weighting behavior in different spatial regions. The thermal modality tends to receive relatively higher weights around the human target region, indicating that thermal information provides stronger local responses associated with human targets under smoke interference. In contrast, infrared information appears to contribute more strongly in some background and structural regions, where richer contour and texture cues are preserved. This qualitative tendency is consistent with the complementary characteristics of the two modalities in smoke-filled environments: thermal imaging provides human-related thermal radiation cues, while infrared imaging preserves more structural and contextual information.

Therefore, Figure 7 provides an intuitive explanation of how the proposed fusion strategy combines complementary information from infrared and thermal modalities. By adaptively balancing the contributions of infrared and thermal modalities, the LVAF module helps the fused image retain both target saliency and background discriminability for subsequent human target detection.

#### 3.6.3. Precision–Recall Curve Analysis

**Figure 8:**
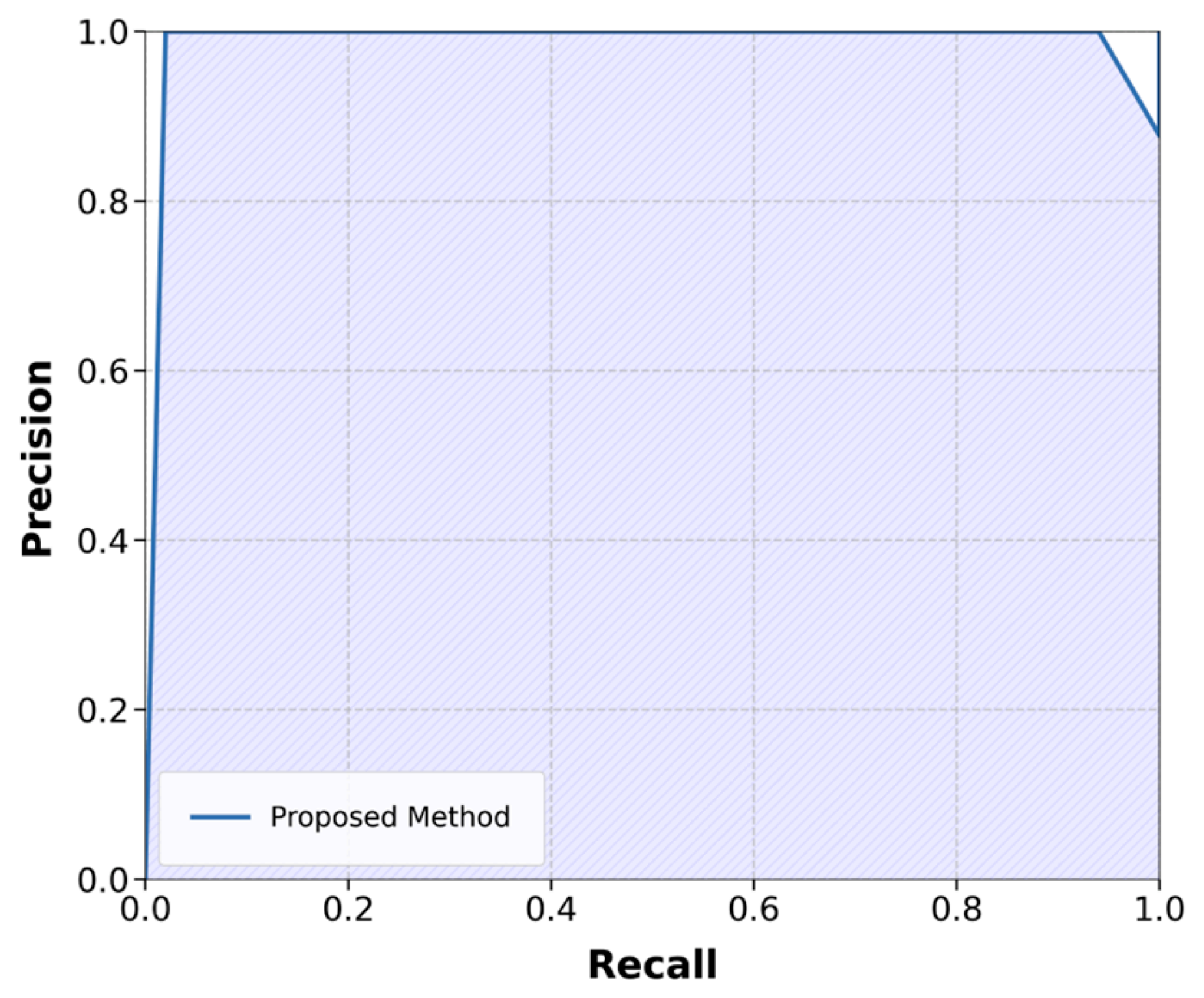
Precision–recall curve of the proposed method on the extended validation set.

Figure 8 shows the Precision–Recall (PR) curve of the proposed method on the extended validation set. The corresponding AP@0.5 reaches 0.9950, which is consistent with the quantitative results reported in Table 1 and Table 2. The PR curve remains close to the upper-right region, indicating that the proposed method maintains high precision over a wide range of recall values.

As recall increases, precision remains at a relatively high level over most of the recall range and decreases gradually only at high recall levels. This indicates that the proposed method maintains a favorable precision–recall balance under different confidence thresholds. Such behavior is particularly important for firefighting rescue scenarios, where maintaining high recall helps reduce missed detections, while sufficient precision helps avoid excessive false alarms.

Therefore, the PR curve provides additional evidence that the proposed method achieves stable detection performance under smoke interference.

## 4. Conclusions

To address the challenges of human target detection in smoke-filled environments, this study proposes an infrared–thermal image fusion detection framework that integrates smoke-oriented infrared enhancement, local variance-guided adaptive fusion, and lightweight human target detection based on YOLO11n. The proposed framework aims to improve target visibility, exploit the complementary information of infrared and thermal modalities, and achieve a favorable balance among detection performance, model complexity, and detector-stage inference efficiency.

First, an improved dark channel prior-based desmoking method is introduced to enhance infrared images degraded by dense smoke. Guided filtering is used to refine the transmission map, and a minimum transmission threshold is incorporated to reduce halo artifacts and over-enhancement. The enhanced infrared images show clearer contours and improved visual contrast, which improves the interpretability of the infrared input and provides an enhanced infrared representation for subsequent multimodal fusion.

Second, a local variance-guided adaptive fusion strategy is developed to integrate desmoked infrared images and thermal images. The fusion module assigns spatially adaptive modality weights according to local saliency information, enabling the fused image to preserve both thermal target saliency and infrared structural details. The ablation results indicate that infrared–thermal multimodal fusion contributes to improved strict localization performance. Compared with the No Fusion configuration, the Proposed Method improves mAP@0.5:0.95 from 0.9205 to 0.9577 and increases Recall from 0.9958 to 0.9989. Compared with fixed-weight fusion, the proposed local variance-guided adaptive fusion strategy maintains comparable detection accuracy while achieving a better detector-stage speed–accuracy balance.

Third, a lightweight YOLO11n detector is adopted as the final detection component. Experimental results show that the YOLO11n-based detector achieves an mAP@0.5 of 0.9950 with only 2.58 M parameters. Compared with larger YOLO-based detectors, the adopted YOLO11n detector maintains comparable detection accuracy with substantially lower model complexity, indicating its suitability as a lightweight detector within the proposed framework. The detector-stage inference speed reaches 73.07 FPS under a batch size of 1 on the tested hardware platform.

Overall, the proposed method does not simply optimize a single metric, but provides a balanced solution in terms of detection accuracy, recall, model complexity, and detector-stage inference efficiency. The experimental results demonstrate that multimodal fusion is essential for reliable human localization under smoke occlusion, while the lightweight detection design provides potential for efficient inference. When considering the complete preprocessing and detection pipeline, the overall latency is approximately 128 ms, while the detector itself achieves 73.07 FPS under batch size 1 inference, indicating its potential applicability to resource-constrained firefighting and rescue robotic platforms in future deployment scenarios.

Nevertheless, this study still has some limitations. First, the current experiments are conducted on a specific dense-smoke infrared–thermal dataset, and further validation on larger-scale datasets with more diverse smoke densities, target distances, and rescue scenarios is required. Second, under extremely dense smoke conditions, the infrared enhancement module and thermal sensing modality may provide insufficient target information due to severe attenuation and occlusion, which may lead to missed detections or localization degradation. Third, the current framework is mainly evaluated in single-person and limited multi-person scenarios. In complex rescue environments containing multiple overlapping targets, severe occlusion, or crowded scenes, further improvements in multi-target association and instance-level perception are needed. In addition, future work will focus on deploying the proposed framework on embedded platforms such as Jetson-series devices and optimizing the preprocessing and fusion modules to achieve more efficient end-to-end inference. Furthermore, lightweight implementations of the LVAF module will be investigated to reduce fusion overhead and improve its suitability for resource-constrained edge deployment.

## Figures and Tables

**Table 1:** Performance comparison of different YOLO-based detectors.

Method	mAP@0.5	mAP@0.5:0.95	Precision	Recall	F1	FPS	Number of Parameters (M)
YOLOv8n	0.9950	0.9583	0.9992	0.9989	0.9991	84.98	3.01
YOLOv8s	0.9950	0.9610	0.9996	0.9987	0.9991	69.48	11.13
YOLO11s	0.9950	0.9580	0.9992	0.9989	0.9991	77.65	9.41
YOLO11m	0.9950	0.9604	1.0000	0.9989	0.9995	57.43	20.03
Proposed Method (YOLO11n)	0.9950	0.9577	0.9998	0.9989	0.9994	73.07	2.58

**Table 2:** Ablation results of preprocessing and fusion strategies.

Configuration	Desmoking	Fusion Strategy	mAP@0.5	mAP@0.5:0.95	Precision	Recall	FPS
Full Method	✓	LVAF Fusion	0.9950	0.9577	0.9998	0.9989	72.79
No Desmoking	✗	LVAF Fusion	0.9950	0.9578	0.9992	0.9989	47.44
No Fusion	✓	Single Modality	0.9949	0.9205	0.9989	0.9958	67.43
Simple Fusion	✓	Fixed Weights	0.9950	0.9562	0.9995	0.9989	51.16

## Data Availability

The experimental dataset used in this study is a self-built dataset, available at the following link: https://doi.org/10.5281/zenodo.20374422.
